# Absence of the Appendix and Ileo-Caecal Valve

**Published:** 1913-02

**Authors:** 


Absence of the Appendix and Ileo-Caecal Valve.—Ducos and Poey-Noguez, *Jour. de Med. de Bourdeaux,* September 20, 1912, describe a case found post-mortem and allude to literature. They have however found no other instance in which the latter was also absent.

